# Crystal Structure of Two Anti-Porphyrin Antibodies with Peroxidase Activity

**DOI:** 10.1371/journal.pone.0051128

**Published:** 2012-12-11

**Authors:** Victor Muñoz Robles, Jean-Didier Maréchal, Amel Bahloul, Marie-Agnès Sari, Jean-Pierre Mahy, Béatrice Golinelli-Pimpaneau

**Affiliations:** 1 Departament de Química, Universitat Autònoma de Barcelona, Edifici C.n., 08193 Cerdanyola del Vallès, Barcelona, Spain; 2 Laboratoire d’Enzymologie et Biochimie structurales, CNRS, Centre de Recherche de Gif, Gif-sur-Yvette, France; 3 Laboratoire de Chimie et Biochimie Pharmacologiques et Toxicologiques, CNRS, Université Paris Descartes, Paris, France; 4 Institut de Chimie Moléculaire et des Matériaux d'Orsay, CNRS, Laboratoire de Chimie Biorganique et Bioinorganique, CNRS, Bât 420, Université Paris 11, Orsay, France; Institut Pasteur, France

## Abstract

We report the crystal structures at 2.05 and 2.45 Å resolution of two antibodies, 13G10 and 14H7, directed against an iron(III)-αααβ-carboxyphenylporphyrin, which display some peroxidase activity. Although these two antibodies differ by only one amino acid in their variable λ-light chain and display 86% sequence identity in their variable heavy chain, their complementary determining regions (CDR) CDRH1 and CDRH3 adopt very different conformations. The presence of Met or Leu residues at positions preceding residue H101 in CDRH3 in 13G10 and 14H7, respectively, yields to shallow combining sites pockets with different shapes that are mainly hydrophobic. The hapten and other carboxyphenyl-derivatized iron(III)-porphyrins have been modeled in the active sites of both antibodies using protein ligand docking with the program GOLD. The hapten is maintained in the antibody pockets of 13G10 and 14H7 by a strong network of hydrogen bonds with two or three carboxylates of the carboxyphenyl substituents of the porphyrin, respectively, as well as numerous stacking and van der Waals interactions with the very hydrophobic CDRH3. However, no amino acid residue was found to chelate the iron. Modeling also allows us to rationalize the recognition of alternative porphyrinic cofactors by the 13G10 and 14H7 antibodies and the effect of imidazole binding on the peroxidase activity of the 13G10/porphyrin complexes.

## Introduction

Hemoproteins contain iron-protoporphyrin IX or heme as the prosthetic group, whose divalent iron atom can reversibly bind molecules such as molecular oxygen, leading to a wide range of biological functions [1]. Chemical or biotechnological models of hemoproteins have thus long been developed in order to create selective catalysts for industrial and fine chemistry and to predict the oxidative metabolism of new drugs [2], [3], [4], [5]. Examples include the *de novo* design of heme proteins, including that of membrane-soluble proteins [6], [7]. Peroxidases appear to be the easiest hemoproteins to be mimicked. Indeed, their active site consists of the iron(III)-porphyrin moiety encapsulated in the apoprotein. On one side, the heme iron is bound to an axial histidine residue (proximal ligand) and on the other side to the peroxide substrate to lead to an iron-oxo complex. The radical cation on the iron (IV)-oxo porphyrin ring can be delocalized onto proximal protein side chains [8]. The reducing cosubstrate does not bind to a well-defined site on the inside of the protein, as peroxidases restrict access of substrates to the heme-oxo complex, so that the electron transfer occurs to the *meso* edge of the heme [9]. Heterolytic cleavage of the O-O bond is assisted by general acid base catalysis through the concerted action of the distal histidine and arginine residues [10]. A major problem in homogeneous metalloporphyrin systems mimicking hemoproteins is that the catalyst is often destroyed by oxidation during the course of the reaction and it is difficult to combine reactivity and selectivity in these models. The use of a protein such as xylanase A [11] or an antibody mimicking the protein matrix of heme enzymes not only prevents aggregation and intermolecular self-oxidation of the catalyst, but can also influence the selectivity of the reaction [12]. As the antibody has the role of a host molecule that enhances the function of porphyrin, the porphyrin itself can be used as the hapten to induce the antibodies.

**Figure 1:**
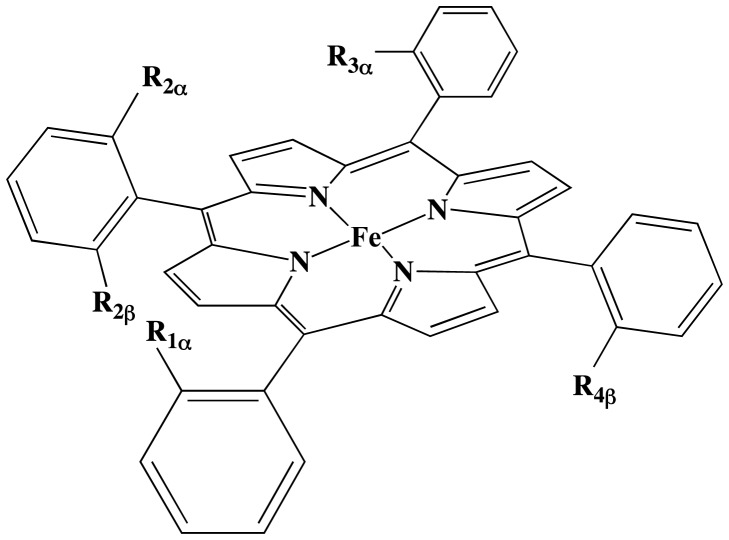
General structures of the cofactors used in this work. R_1α_ = R_2α_ = R_3α_ = R_4β_ = COOH, R_2β_ = H: iron(III)- α,α,α,β-*mesotetrakis*-orthocarboxyphenyl-porphyrin Fe(ToCPP) hapten used to induce the antibodies 13G10 and 14H7; R_1α_ = R_2α_ = COOH, R_2β_ = R_3α_ = R_4β_ = H: αα-Fe(DoCPP); R_1α_ = R_2β_ = COOH, R_2α_ = R_3α_ = R_4β_ = H: αβ-Fe(DoCPP); R_1α_ = COOH, R_2α_ = R_2β_ = R_3α_ = R_4β_ = H: Fe(MoCPP).

In order to generate antibodies with peroxidase activity, mice have been immunized against iron(III)-α,α,α,β-*meso-*tetraki*s*-orthocarboxyphenyl-porphyrin (Fe(ToCPP)) (Figure 1) [13], [14]. Two antibodies, 13G10 and 14H7, were found to bind the porphyrin hapten with nanomolar affinities and enhance its peroxidase activity. The 13G10-Fe(ToCPP) and 14H7-Fe(ToCPP) complexes catalyzed the oxidation of 2,2′-azinobis (3-ethylbenzothiazoline-6-sulfonic acid) (ABTS) 5 to 8 fold more effectively than the cofactor alone, with k_cat_ reaching 540 min^−1^ and k_cat_/K_m_^(ABTS)^ 6.2 10^4^ M^−1^ min^−1^ at pH 4.6 for the 13G10 complex [15]. The antibodies protected the cofactor from oxidative degradation, allowing more than one thousand turnovers before destruction. In addition, it was shown that the 13G10-Fe(ToCPP) complex possessed a remarkably thermostable peroxidase activity and that it was able to use not only H_2_O_2_ as the oxidant but also a wide range of hydroperoxides [15], [16]. Compared with the peroxidase enzymes, the two antibodies possess a low affinity toward H_2_O_2_ (*K_m_* = 9–16 mM) but a higher affinity than the cofactor alone (*K_m_* = 42 mM) [13]. Because no amino acid was found to coordinate the iron atom in the 13G10 and 14H7/Fe(ToCPP) complexes [14], it was thought that an imidazole ligand could mimic the proximal histidine of peroxidases and enhance the catalytic activity. Fe(ToCPP) and less hindered iron(III)-tetraarylporphyrins, bearing only one or two carboxyphenyl substituents, were found to bind only one imidazole ligand when complexed to antibody 13G10, suggesting that the complexes are good mimics for peroxidases [16]. Whereas 50 mM imidazole inhibited the peroxidase activity of the 13G10-Fe(ToCPP) complex, it increased that of 13G10 in complex with porphyrins bearing two *meso*-[ortho-carboxyphenyl] substituents by a factor 15.

To get insight into the structural basis for the different binding/activities of the variously substituted porphyrins, we have determined the crystal structure of the fragment antigen binding (Fab) of antibodies 13G10 and 14H7 and modeled their interaction with the different iron-porphyrins using molecular docking.

## Materials and Methods

### Purification and Characterization of Fabs 13G10 and 14H7

The antibodies were produced in ascitic fluid as described [13]. After ammonium sulfate precipitation, the IgGs (IgG1, λ) were loaded on a protein A column in 3 M NaCl, 1.5 M glycine pH 8.9 and eluted by 0.1 M citrate pH 5. The Fab 13G10 (resp. 14H7) was generated by papain digestion of the antibody at 37°C under standard conditions (30 mM Tris pH 7.4, 138 mM NaCl, 1.25 mM EDTA, 1.5 mM 2-mercaptoethanol) using a 3% papain to antibody ratio (w/w) and a 10 h (resp. 8 h) digestion time. Undigested IgG and Fc fragment were removed by DEAE anion exchange chromatography followed by gel filtration on a Sephacryl S100 HR column. The Fabs were further purified by ion exchange chromatography on a mono Q FPLC column by a NaCl gradient in 20 mM bistrispropane buffer at pH 7.2 for Fab 14H7 or in 20 mM diethanolamine buffer at pH 8.8 for Fab 13G10.

### Sequence Determination

N-terminal sequencing of the H chain of 13G10 showed that the first three amino acid residues of the Fab were missing. mRNA from 13G10 hybridoma cells (4 10^6^ cells) were isolated using a mRNA purification kit from Dynal. cDNAs were prepared in 50µl using the Omniscript Reverse Transcriptase kit from Qiagen and stored at −20°C until use. The heavy chain variable region fragment (about 420 bp) of each cDNA was amplified using a set of 12 forward primers MHV1 to MHV12 previously described [17] and reverse primer IgG1∶ 5′GGATCCCGGGCCAGTGGATAGACAGATG complementary to the beginning of the constant region. The λ-light chain fragment (about 690 bp) of each cDNA was amplified using a mixture of 2 forward primers, NL1∶ 5′ATGGCCTGGATTTCACTTATAC and NL2∶ 5′ATGGCCTGGACTCCTCTCTTC, corresponding to mouse λ-light chain leader sequence, and a mixture of 4 reverse primers, CL1 5′GCAGGAGACAGACTCTTCTCCAC, CL2 5′GCACGAGACAGACTCTTCTCCAC, CL3 5′GCAGGGGACAAACTCTTCTCCAC, and CL4 5′GCACGGGACAAACTCTTCTCCAC, complementary to mouse CLterminal sequence.

Each cDNA mixture (3 µl) was amplified with the Polymerase Chain Reaction [18] using TFL polymerase (Promega) on a MJ Research mini cycler with the following programm. A 10 min denaturation step at 94°C was followed by 30 cycles of 30 sec denaturation at 94°C, 45 sec hybridization at 59°C and 1 min 30 sec elongation at 72°C, a final step of 10 min at 72°C was performed to ensure completion of the amplification. Amplified products were purified on 0.8% low melting agarose (Sigma) and ligated into pGEM®Teasy vector (Promega). Electrocompetent *E. coli* TG1 cells {D(lac pro) supE thi hsdD5 F' traD35 proAB LacIq LacZDM15} were transformed with the ligation mixture by electroporation using a Cell porator electroporation system equipped with a Voltage Booster (Life technologies) according to manufacturer's recommendations. Plasmid DNAs were extracted from transformed cells and submitted to dideoxy sequencing [19]. For each antibody, clones were originated from at least two independant Polymerase Chain Reactions.

### Crystallization

Crystals of 13G10 were grown in 26.5% PEG 2000, 0.2 M MgCl_2_, 0.1 M Tris pH 8.5, 10% glycerol. Crystals were flash cooled in a nitrogen stream at 100 K in the same solution containing 10% glycerol. Crystals of 14H7 were grown in 25% PEG 4000, 0.1 M ammonium acetate, 0.1 M sodium cacodylate pH 6.5. A single capillary-mounted crystal kept at 4°C was used for data collection. This explains the low redundancy for the 14H7 data.

#### X-Ray data collection and structure determination

Diffraction data for Fab 13G10 and Fab 14H7 were recorded at the ID14-1 station of ESRF and the LURE DW32 station, respectively. Data were processed with DENZO and SCALEPACK [20] (Table 1). The structures were solved by molecular replacement with the program AMoRe [21]; the models used were the Fv domain (PDB code 1mfa) and the CL-CH1 dimer (PDB code 1mfe) of the murine anticarbohydrate antibody Se155-4, which belongs to the same IgG1, λ class [22]. The atomic model of 13G10 was refined alternating cycles of model reconstruction with *O* [23] and refinement with *CNS* [24], whereas the atomic model of 14H7 was refined alternating cycles of model reconstruction with *COOT* and refinement with *PHENIX* [25] using the twin option. The final refinement statistics are given in Table 1. Several residues at the N-terminus of the heavy chain and CDRH1 of four heavy chains that were disordered were not included in the model. Figures were drawn with *PYMOL*. ThrL51 in CDRL2, which lies in the disallowed region of the Ramachandran plot, belongs to a γ turn, as commonly observed in all antibody structures.

**Table 1:** Data collection and refinement statistics for Fab 13G10 and 14H7.

Data collection	Fab13G10	Fab14H7
Space group	C2	P21
Number of molecules in the asymmetric unit	1	8
unit cell	a = 55.8 Å, b = 62.7 Å, c = 113.5 Å	a = 56.2 Å, b = 228.2 Å, c = 146.6 Å
	α = 90, β = 91.7°, γ = 90°	α = 90, β = 90.1°, γ = 90°
resolution	25–2.05 Å	20–2.55 Å
(outer resolution shell)	2.09–2.05 Å	2.64–2.55 Å
unique reflections	23580	111576
completeness*	93.7% (91.4%)	93.7% (87.8%)
mean I/sigma*	23.7 (4.6)	7.4 (2.1)
R_sym_ *	0.037 (0.19)	0.109 (0.51)
redundancy*	2.5 (2.1)	1.2 (1.2)
Refinement statistics		
resolution	20.0–2.05	20.0–2.45
protein residues	425	3356
water molecules	188	359
glycerol molecules	3	–
Mg^2+^ molecule	1	–
R_cryst_	0.224	0.279
R_free_ ‡	0.275	0.328
Deviations from ideal geometry (rms)		
bond length deviation (Å)	0.008	0.003
bond angle deviation (°)	1.52	0.75
B values		
Average B value of protein atoms (Å^2^)	49.0	46.5
Average B value of water molecules (Å^2^)	51.1	25.5
Average B value of glycerol (Å^2^)	76.3	–
B value of Mg^2+^ (Å^2^)	45.0	–
Ramachandran plot		
Most favored (%)	86.1	79.6
Additionally allowed (%)	11.4	17.5
Generously allowed (%)	1.1	1.4
Disallowed (%)	1.4	1.5

* Values for highest-resolution shell are given in parentheses.

‡ 4.5% and 4.7% of the data were set aside for the R_free_ calculation during the entire refinement.

### Molecular Modeling

Quantum mechanical calculations were carried out to model the structures of the tetra-, bi- and mono substituted porphyrins. These structures were fully optimized with the density functional B3LYP [26], [27], as implemented in Gaussian09 [28]. The double-z basis set LANL2DZ [29] and its associated pseudo potential were used for the iron and the split valence 6–31 g** [30], [31] for the other atoms (C, N, H and O). Calculations were carried out for the high spin ferric species with the iron coordinated by the four porphyrin nitrogen atoms. The structures of the 13G10 and 14H7 antibodies were processed with the UCSF Chimera package [32] prior to docking in order to remove the water, ions and glycerol molecules, calculate the protonation states of the amino acids and add hydrogen atoms. Protein-ligand dockings were undertaken for both antibody structures following a recently published protocol that accounts for the presence of the metal in the ligand [33]. Calculations were carried out using the program GOLD (version 5.1) [34] and the Chemscore [35] scoring function. The metal ion was modeled thanks to an *in house* parameterization of an additional atom type in GOLD, which mimics the chelating capacity of the metal by interacting with amino acids with potential Lewis basis capacities. For the docking of imidazole, the parameters of the Chemscore scoring function used for the iron atom were that of cytochrome P450, as implemented in GOLD5.0. Because the Mg^2+^ ion lies at the interface between the hypervariable loops in the structure of 13G10 and probably mimics the iron of the porphyrin hapten, the docking calculations were performed using a 20 Å sphere around the Nδ atom of the neighboring residue AsnH33. Based on initial rigid dockings, full flexibility was allowed for TyrL34 using the Dunbrack rotamer library [36]. The bonds between the carboxyphenyl groups and the porphyrin moiety were frozen to maintain the correct α/β configuration. For each docking, an *in house* approach, based on a statistical analysis of a large PDB set of metal-bound proteins, was used in order to identify those residues that could directly bind to the metal. Based on the distances between the metal and the Cα carbons of all neighboring residues, no direct interaction was identified between the metal and the protein. Therefore, no further QM/MM refinements of the complexes were carried out [33].

## Results

### Amino Acid Sequence of Fab 13G10 and Fab 14H7

**Figure 2:**
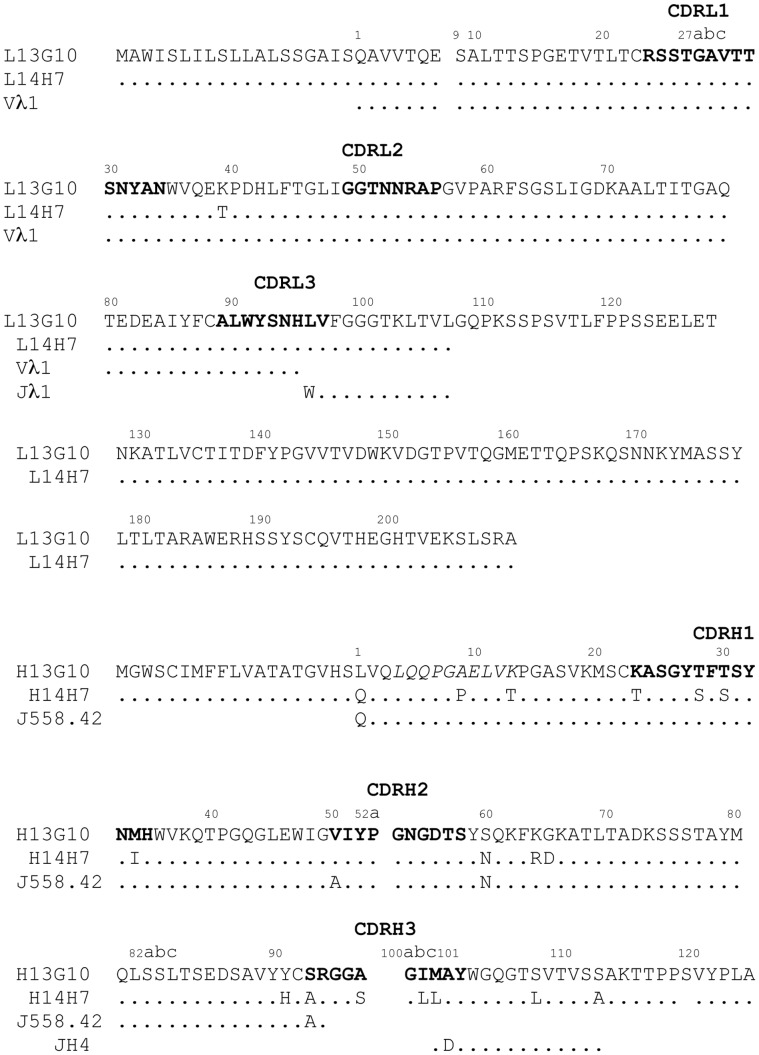
Amino acid sequences of the VL and VH domains of 13G10 and 14H7. Dots denote sequence identity to antibody 13G10. H denotes the heavy chain and L the light chain. Numbering of the antibody residues follows the Kabat nomenclature [40] and the definition of the hypervariable regions, indicated in bold, is from North et al. [37]. The sequence in italics has also been determined by Edman degradation of the aminoterminal part of the protein. The nucleotide sequences of 13G10 and 14H7 have been deposited in the Genbank database, except for the constant heavy chains whose sequences have not been determined: Genbank accession numbers 13G10H, **AY178830**; 13G10L, **AY178831** for the mRNA and **AA020092**, **AA020093** for the corresponding protein sequence; 14H7H, **AY178829**; 14H7L, **AY178828** for the mRNA and **AA020092**, **AA020093** for the corresponding protein sequence. The previously published amino acid sequences [14] were not those of antibodies 13G10 and 14H7. The germline sequences are indicated below the sequence of the antibodies.

13G10 and 14H7 are murine monoclonal antibodies that were found to belong to the IgG1, λ-class. The sequences of their variable chains (Figure 2) differ by one residue in the light chain, outside of the complementary determining regions (CDRs) [37], and 17 residues in the heavy chain. In CDRH1, the sequences differ at positions H23 (Lys/Thr), H28 (Thr/Ser), H30 (Thr/Ser) and H34 (Met/Ile) for 13G10 and 14H7, respectively. CDRH2 has the same sequence in both antibodies. Their CDRH3, which is a main determinant of the combining site, has the same length but contains, in addition to Ser/Ala mutations at positions 93 and 97, two different amino acids with the same hydrophobic nature at position H100b (Ile/Leu) and H100c (Met/Leu).

Mature antibody genes are first formed by the assembly of the variable (V) and junction (J) gene segments for the light chain variable (VL) domain and by the assembly of the variable (V), junction (J) and diversity (D) gene segments for the heavy chain variable (VH) domain; affinity maturation then introduces into antibodies somatic mutations that increase binding affinity to the hapten or antigen [38]. For mice (*Mus Musculus*) antibodies, only three germline Vλsegments (compared with 180 Vκ segments), and four Jλ segments have been uncovered [39], which explains the poor diversity of the λ-light chain variable sequences compared with the κ-class. Compared with the germline Vλ1/Jλ1 sequence [40], there is one difference in the variable light chain of 13G10 and 14H7, Leu at position L96 (instead of Trp) in CDRL3 that arises from diversity at the junction between the V and J gene segments (Figure 2). In addition, 14H7 displays a Thr instead of a Lys at position L39, which is located outside the recombinant site. The nucleotide sequence of the 13G10 and 14H7 VH domains shows that they are derived from a gene in the J558 family, the largest VH family. The 13G10 VH sequence has highest homology to germline gene sequence J558.42 (98% identity at the nucleotide level) [41]. This translates into 94 (resp. 87) identical amino acid residues out 98 in VH for 13G10 and 14H7, respectively (Figure 2). The nucleotide sequence indicates that the VH region of antibodies 13G10 and 14H7 is encoded by the combination of J558.42 with a D gene segment that could not be identified because it is too short and JH4.

Comparison of the 13G10 and 14H7 VL and VH sequences to that of the germline genes indicates that 13G10 and 14H7 did not undergo much affinity maturation in the V segments. Among the rare somatic mutations, the ones that belong to the combining sites are LeuL96 (CDRL3), ValH50 (CDRH2) and SerH93 (CDRH3), for 13G10 (Table 2, Figure 2). On the other hand, the exceedingly hydrophobic nature of CDRH3 of 13G10 and 14H7 is due to extensive affinity maturation in the D segment so that the germline D segment could not be identified from the nucleotide sequence. Alanine at position H101 comes from a somatic mutation.

**Table 2:** Comparison of the aminoacids that contact small haptens in the structurally characterized λ-light chain antibodies.

PDB code		13G10/14H7*	Se155-41mfa [22]	CHA2551ind [79]	NC101etz [83]	88C6/121yuh [80]	2D12.51nc2 [85]	RS2-G192ntf [82]	10G61jnh [84]	N1G91ngp [81]
**L32**	CDRL1	**Tyr**	**His**	Tyr	Tyr	**Tyr**	**Tyr**	**Tyr**	Tyr	**Tyr**
**L34**	–	Asn	Asn	Asn	Ile	Asn	Asn	**Asn**	Asn	Asn
**L91**	CDRL3	**Trp**	**Trp**	**Trp**	**Trp**	**Trp**	**Trp**	**Trp**	**Trp**	**Trp**
**L93**	–	Ser	**Asn**	**Ser**	Ser	Ser	Ser	Ser	Ser	Ser
**L94**	–	**Asn**	**Asn**	Asn	Asn	Asn	Asn	Asn	**Asn**	Asn
**L96**	–	**Leu**	**Trp**	**Trp**	**Trp**	**Trp**	**Trp**	**Trp**	**Phe**	**Trp**
**H33**	CDRH1	**Asn**	**Trp**	**Thr**	Gly	**Leu**	Gly	**Trp**	**Trp**	**Trp**
**H35**	–	**His**	**His**	Ser	Gly	**His**	**His**	**His**	**Gln**	**His**
**H47**	FR2	Trp	Trp	Trp	Leu	Trp	Trp	Trp	**Trp**	Trp
**H50**	CDRH2	**Val**	**Ala**	**Thr**	**Asp**	**Arg**	Val	Thr	**Ala**	**Arg**
**H52**	–	**Tyr**	Tyr	**Leu**	**Trp**	**Asp**	**Trp**	Tyr	Tyr	Asp
**H52A**	–	Pro	Pro	**Ser**		Pro		Pro	Pro	Pro
**H53**	–	Gly	Asn	Gly	Asn	Asn	**Ser**	Gly	Gly	Asn
**H56**	–	**Asp**	**Ala**	**Phe**	**Lys**	Val	Gly	Asn	Asp	Gly
**H58**	–	**Ser**	**Phe**	**Phe**	**Tyr**	**Lys**	Ala	**Tyr**	**Arg**	**Lys**
**H95**	CDRH3	**Gly**	Gly	**His**	**Arg**	**Tyr**	**Arg**	Gly	**Gly**	**Tyr**
**H96**	–	**Gly**	**Gly**	Arg	Thr	**Ala**	**Gly**	**Ser**	**Arg**	Asp
**H97**	–	**Ala/Ser**	**His**		Phe	**Tyr**	**Ser**	Leu	**Ser**	**Tyr**
**H98**	–				Ser	**Cys**	**Tyr**	Tyr	**Leu**	Tyr
**H99**	–				Tyr	**Arg**	Pro	Tyr	Tyr	Gly
**H100**	–				Tyr		Tyr	Asn		Ser
**H100**	–				Tyr					
**H100**	–				Gly					
**H100**	–				Ser					
**H100**	–				Ser			**Asn**		
**H100**	–		**Gly**		Phe			Tyr		
**H100**	–	**Gly**	**Tyr**		**Ty**r		**Asn**	Gly	Tyr	**Ser**
**H100**	–	Ile	Tyr		Tyr	Pro	Tyr	Trp	**Thr**	Tyr
**H100**		Met/Leu	Gly		Phe	Met	Phe	**Phe**	Met	Phe
**H101**		Ala	Asp	Val	Asp	Asp	Asp	Gly	Asp	Asp

Underligned letters are residues that form direct or water-mediated hydrogen-bonds or salt-bridge to the hapten. A bold letter indicates that the residue is in van der Waals contacts with the hapten.

* For 13G10 and 14H7, the hapten has been modeled by docking (see Table 3).

### Determination of the Structures of Fab 13G10 and Fab 14H7

The crystal structures of Fab 13G10 and Fab 14H7 were determined at 2.05 Å and 2.55 Å, respectively (Table 1). Fab 13G10 crystallized with one molecule in the asymmetric unit, whereas the crystals of Fab 14H7 contained eight Fab molecules in the asymmetric unit and were pseudomerohedrally twinned [42], [43], [44]. Twinning was detected using the xtriage program included in *PHENIX* [25]. The cumulative local intensity deviation distribution statistics [45] did not indicate twinning: <|L|> = 0.488 (untwinned: 0.500; perfect twin: 0.375); <L^2^> = 0.319 (untwinned: 0.333; perfect twin: 0.200). However, the results of the H-test [46] on acentric data gave a <|H|> value of 0.046 (0.50: untwinned; 0.0∶50% twinned) and a <H^2^> value of 0.005 (0.33: untwinned; 0.0∶50% twinned). The estimation of the twin fraction by the H-test was 0.454 compared with 0.425 by Britton analysis [47] (Figure S1). The twinning law h, -k, -l and a twinning fraction of 0.5 were used throughout the refinement using *PHENIX*.

### Overview of the Structures of Fab 13G10 and Fab 14H7

**Figure 3:**
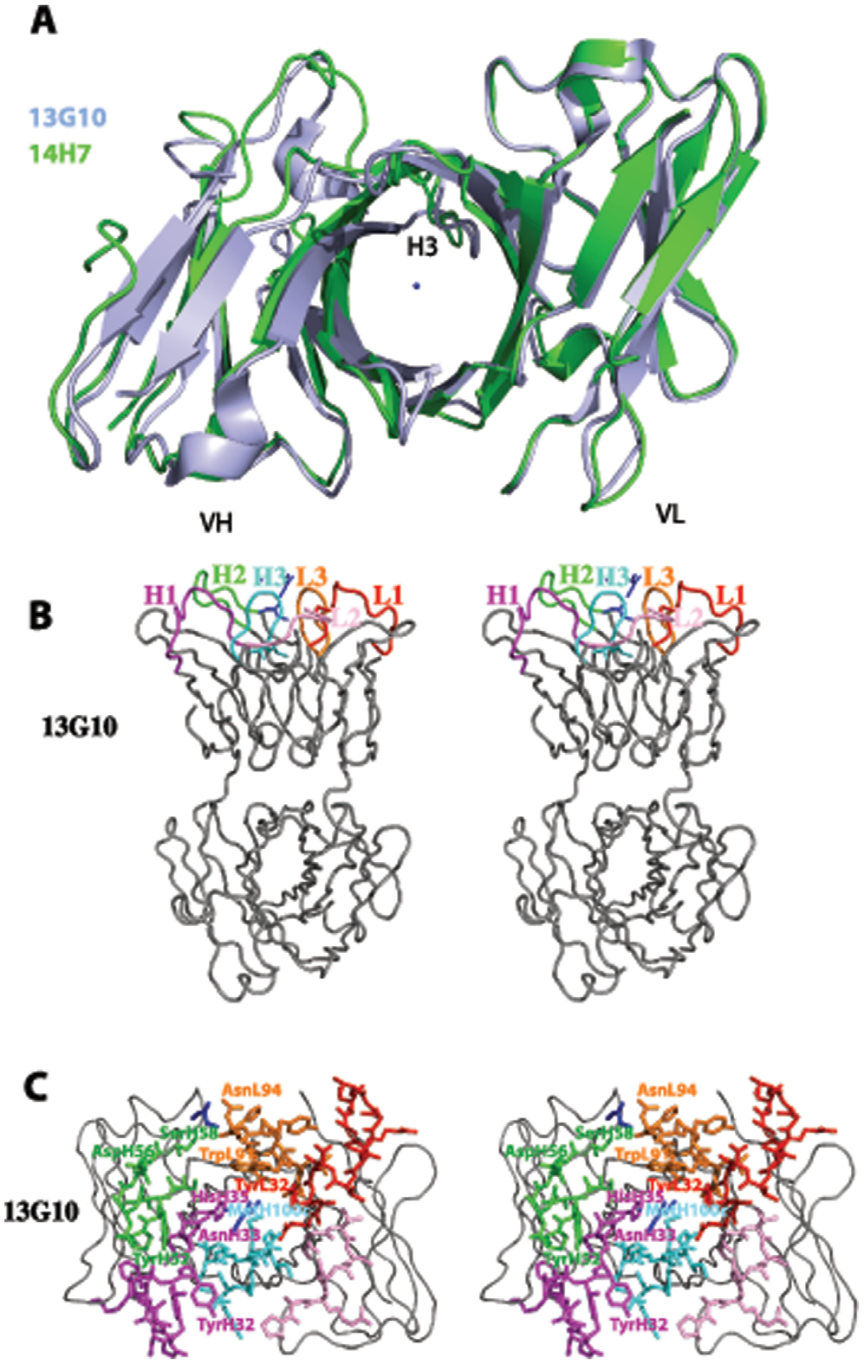
General view of the combining sites of Fab 13G10 and Fab 14H7. **A** Comparison of the binding site cavities of 13G10 and 14H7. The VL frameworks of 13G10 (in blue) and 14H7 (in green) have been superimposed. The location of Mg^2+^ in 13G10 is indicated as a blue ball. **B** General view of the active site of 13G10. CDRL1, CDRL2, CDRL3, CDRH1, CDRH2 and CDRH3 are shown as red, pink, orange, magenta, green and cyan ribbons, respectively. Mg^2+^ and two glycerol molecules are indicated as blue ball and sticks, respectively. **C** Zoom of the combining site of 13G10.

Fab 13G10 and Fab 14H7 display large elbow angles of 175.3° and 170.1°, respectively, in agreement with their light chain belonging to the λ-class. The frameworks of the two Fabs superimpose with an rmsd of 1.067 Å^2^ over 174 Cα atoms. However, the fit is better for the VL and VH domains taken separately, with an rmsd of 0.54 Å^2^ over 89 Cα atoms and 0.92 Å^2^ over 85 Cα atoms, respectively. This reflects a difference in the orientation of the VH and VL domains of the two antibodies with respect to each other of 8.04° and 0.61 Å. Together with the different conformations of CDRH3 (see below), the combining site of 14H7 is more open, with an accessible surface area of 19810 Å^2^ compared with 18820 Å^2^ for 13G10 (Figure 3A).

**Figure 4:**
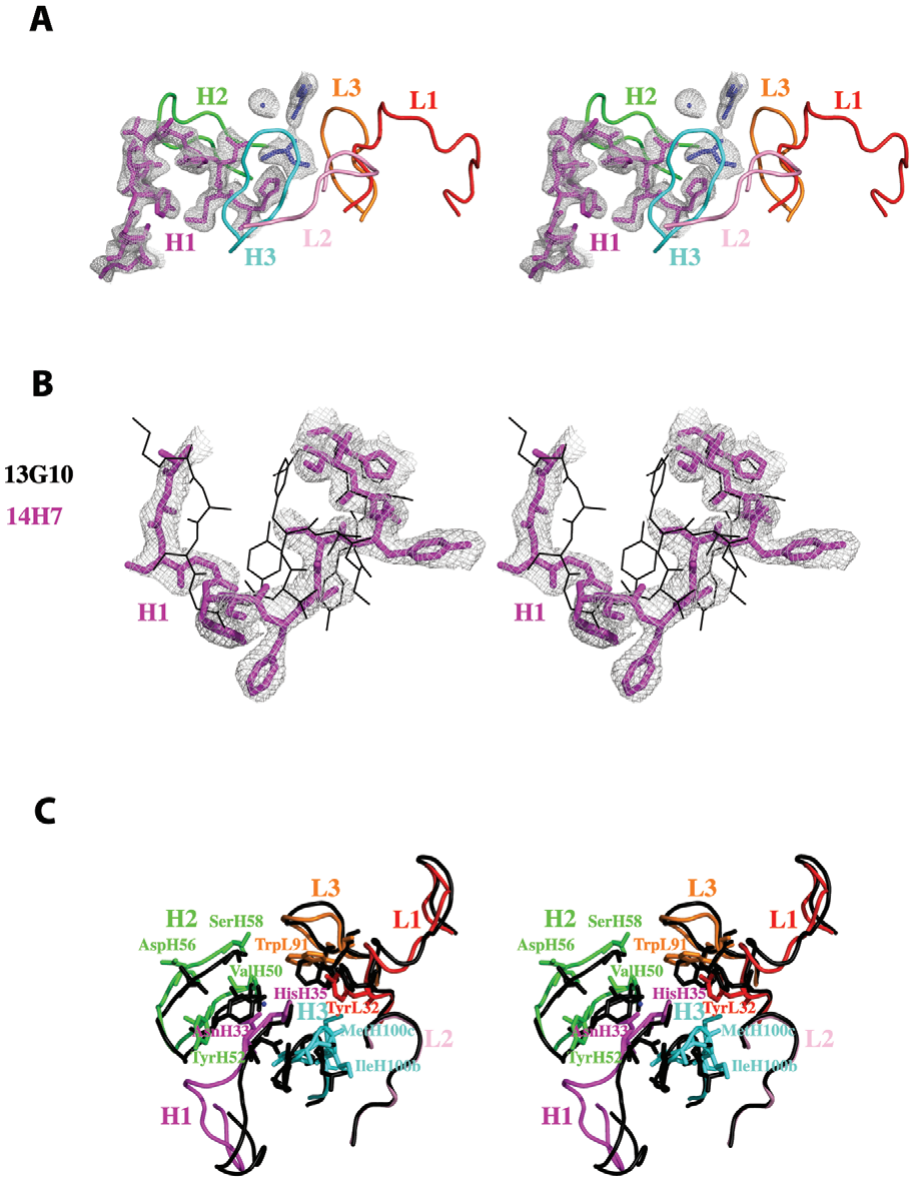
Comparison of the CDRs of Fab 13G10 and Fab 14H7. **A** Peculiar conformation of CDRH1 in the combining site of Fab 13G10. CDRH1 is shown as magenta sticks and a 2Fo-Fcalc map, contoured at 1 σ and displayed around CDRH1, Mg^2+^ and the two glycerol molecules, as a grey mesh. **B** Comparison of the conformations of CDRH1 in 13G10 and 14H7. CDRH1 in 14H7 and 13G10 are shown as magenta sticks and black lines, respectively. The VH frameweworks of the two antibodies were superimposed. A 2Fo-Fcalc map contoured at 1 σ is displayed around CDRH1 of 14H7 and shown as a grey mesh. **C** Comparison of the combining sites of Fab 13G10 (CDRs colored as in Fig. 4A) and Fab 14H7 (black).

The hapten-binding pocket of 13G10 is a shallow cleft, roughly 12.7 Å by 7.2 Å wide and 8 Å deep, at the upper part of the VL/VH interface (Figure 3B and 3C). Electron density could be observed for a magnesium ion and two molecules of glycerol in the combining site of Fab 13G10 (Figures 3B and 4A). The magnesium ion likely indicates the position of the Fe(ToCPP) iron in the hapten/antibody complex, thus confirming the location of the catalytic pocket at the antibody combining site at the VL/VH interface.

In 13G10, CDRH1 adopts the canonical structure H1-13-1 (Figure 4A) [37]. In 14H7, electron density is observed for CDRH1 only for four molecules in the asymmetric unit out of eight and it adopts a very different conformation from that in 13G10, which has not yet been listed (Figure 4B) [37]. This is a consequence of the four differences in amino acids in this CDR between the two antibodies. However, this difference in the Cα backbone of CDRH1 does not contribute to create different topologies in the combining sites because only AsnH33, whose side chain has two different conformations in the two antibodies, together with HisH35, line the pocket (Figure 3C). CDRH2 adopts the canonical structures H2-10-1 in both antibodies [37] but the side chains of TyrH52 display very different conformations (Figure 4C). CDRH3 in both antibodies does not share the same conformation because of the flexibility of the main chain due to the presence of three glycine residues (Figure 2). AsnH33, TyrH52 and the main chain of CDRH3 are in contact so that their conformation depends on each other (Figure 4C). In both 13G10 and 14H7, CDRL1, CDRL2 and CDRL3 adopt the canonical structures L1-14-1, L2-8-4 and L3-9-1, respectively [37]. The side chain of TrpL91 in CDRL3 has different orientations in the two antibodies (Figure 4C).

AsnH33, several residues of CDRH2 (TyrH52, AspH56, SerH58 and the somatically mutated residue ValH50) and CDRH3 form one face of the combining site (Figure 3B and 3C). The main residues lining the other face of the cavity are TyrL32 and TrpL91. The bottom of the site is composed of HisH35 and somatically mutated LeuL96. The identity of the amino acids that define the combining site of 13G10 and 14H7 is consistent with their belonging to the λ-light chain class of antibodies (Table 2). The very hydrophobic nature of the antigen-binding site is not only due to the presence of Tyr, Trp and Leu residues at contact positions, as observed in other antibody structures (Table 2). In addition, the CDRH3 loops of 13G10 and 14H7 are very peculiar in that they contain a high number of glycine and alanine residues (H95-H100a) and only a few residues with a long side-chain that could interact with the hapten (Figure 2).

### Molecular Modeling of the α, α, α, β Hapten-antibody Complexes

**Figure 5:**
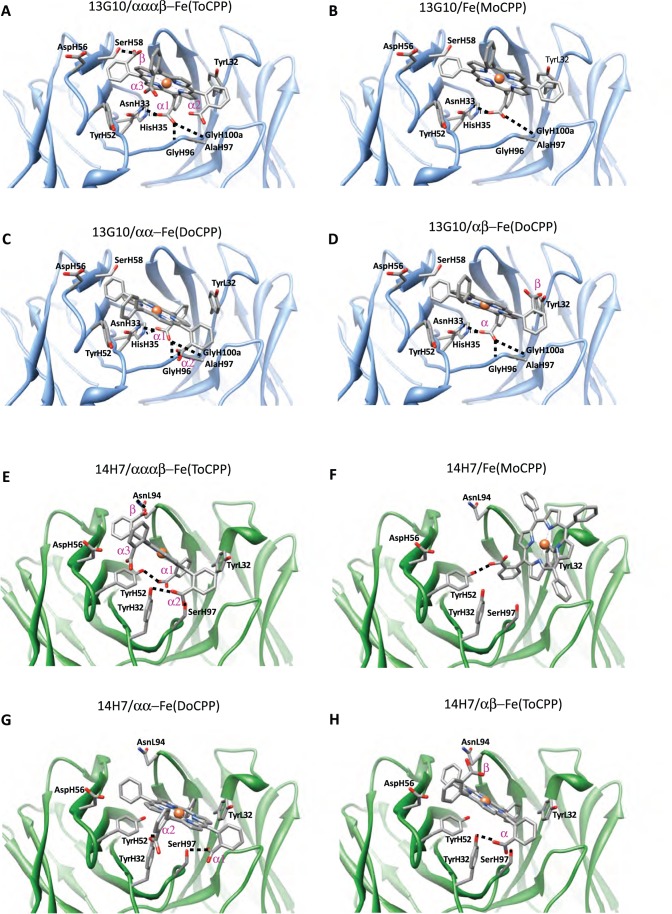
Models of the complexes of 13G10 and 14H7 with the different porphyrins. The H-bonds between the antibodies and the porphyrins are indicated. **A** 13G10/Fe(ToCPP). Molecular docking gives two most favorable orientations of Fe(ToCPP) bound to 13G10. In the most stable 13G10/Fe(ToCPP) predicted complex, the carboxylate of the β substituent makes a H-bond with SerH58, while the α1 substituent is H-bonded to AsnH33, HisH35, GlyH96 and GlyH100a. Only two bad contacts between the ligand and protein are observed in this orientation (Table 3). In the second orientation (score 40.4 compared with 41.5, data not shown), this pair of interactions is inverted. **B** 13G10/Fe(MoCPP). The unique carboxyphenyl substituent is plugged inside the binding site of 13G10, making hydrogen bonds with AsnH33, HisH35 and GlyH100a. One of the phenyl substituents is also inserted into the cavity, while the two others remain solvent exposed. The predicted complex has a similar intermolecular energy than the 13G10/Fe(ToCPP) complex, with no major bad contacts. **C** 13G10/αα-Fe(DoCPP). **D** 13G10/αβ-Fe(DoCPP). For the two Fe(DoCPP) derivatives, one of the carboxyphenyl substituents is anchored in the binding site of 13G10, while the other is exposed to the solvent. The buried carboxylate makes hydrogen bonds with AsnH33, HisH35, GlyH100 and GlyH100a (Table 3). For αα-Fe(DoCPP), the second carboxylate is interacting with AlaH97, while in its αβ counterpart, it does not make any interaction. As a result, the calculated energy of the αα-Fe(DoCPP)/13G10 complex is 2 kJ/mol lower than that of the αβ-Fe(DoCPP)/13G10 complex (Table 3). **E** 14H7/Fe(ToCPP). In the best 14H7/Fe(ToCPP) model, the main hydrogen bonding interactions are formed between the α2 substituent and both SerH97 and TyrH32, and between the α1 substituent and TyrH52 (Table 3). A weaker interaction occurs between the β substituent and AsnL94 and no major bad contacts are found between any residue and the porphyrin cofactor. In other orientations with higher energies, the porphyrin iron is chelated by TyrL32, as observed with Fe(MoCPP). **F** 14H7/Fe(MoCPP). The unique carboxylate substituent is interacting with TyrH52, whereas TyrL32 is chelating the metal atom. **G** 14H7/αα-Fe(DoCPP). **H** 14H7/αβ-Fe(DoCPP). For αα-Fe(DoCPP) and αβ-Fe(DoCPP), the interactions of 14H7 with TyrH32 and SerH97, which were present in the 14H7/Fe(ToCPP) complex, are maintained. Interestingly, in the αβ-Fe(DoCPP)/13G10 complex, the interactions with SerH97 and TyrH32 are made with the same carboxylate group, whereas, in αα-Fe(DoCPP)/13G10, one carboxylate substituent is interacting with TyrH32 and the other with SerH97. This leads to the reduction of bad contacts in the latter case (Table 3).

**Figure 6:**
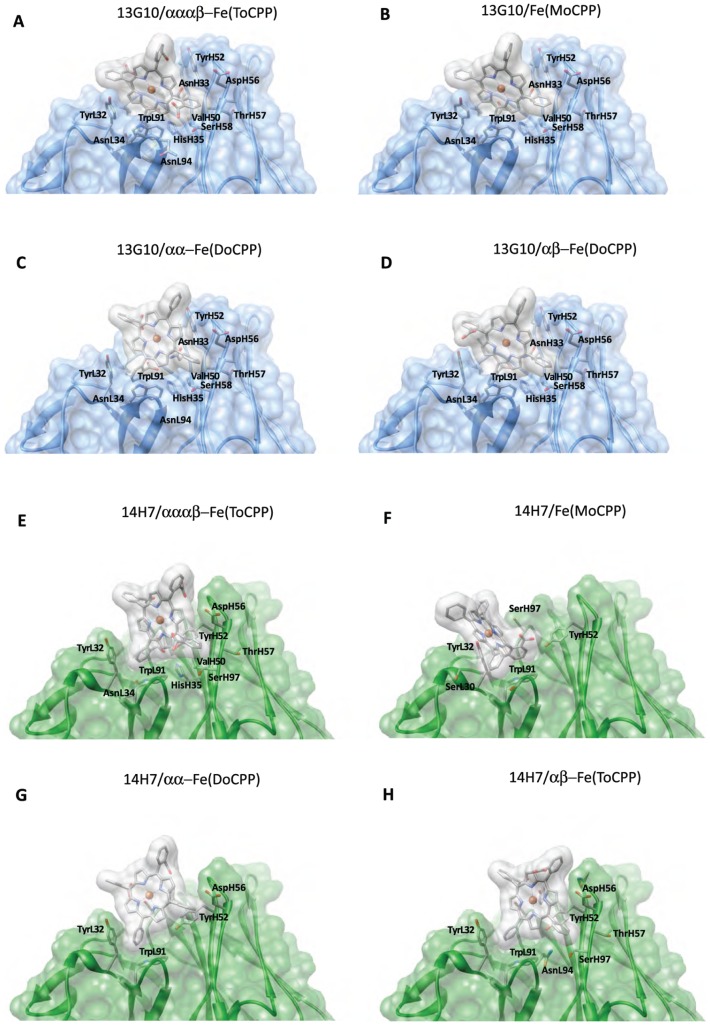
Molecular surfaces of the models of the complexes of 13G10 and 14H7 with the different porphyrins. Residues involved in van der Waals and hydrophobic contacts are shown. **A** 13G10/Fe(ToCPP). **B** 13G10/Fe(MoCPP). **C** 13G10/αα-Fe(DoCPP). **D** 13G10/αβ-Fe(DoCPP). **E** 14H7/Fe(ToCPP). **F** 14H7/Fe(MoCPP). **G** 14H7/αα-Fe(DoCPP). **H** 14H7/αβ-Fe(DoCPP).

Because we did not succeed to grow crystals of Fab 13G10 and Fab 14H7 in complex with the Fe(ToCPP) hapten, either by co-crystallization or soaking, Fe(ToCPP) was modeled in the recombinant site by molecular docking using the Gold 5.0 package [34] and a previously reported protocol [33] (Figures 5A, 5E, 6A and 6E; Table 3). The docking solutions display the adjacent α1 and α2 carboxyphenyl substituents deeply buried inside the binding site, while those in α3 and β configuration are exposed toward the solvent. The comparison of the accessible surface areas of Fe(ToCPP) alone and in complex with 13G10 and 14H7 indicates that 53.7% and 44.9%, respectively, of the porphyrin is buried in the antibody pocket.

**Table 3:** Statistical analysis of the lowest energy structures obtained in the docking calculations of the different cofactors in the 13G10 and 14H7 antibody structures.

Antibody	Ligand	Buried surface area (Å^2^)^1^	Score (kJ/mol)^2^	ΔG (kJ/mol)^2^	S_hbond_ (kJ/mol)^2^	Hydrogen bondingInteractions(distances in Å)	S_lipo_(kJ/mol)^2^	Hydrophobic contacts	S_clash_(kJ/mol)^2^	Clashe (distancesin Å)	S_internal_(kJ/mol)^2^	Iron distance(Å)^3^
13G10	Fe(ToCPP)	119.3	41.49	−49.13	3.38	β-COO^−^/SerH58 OH (2.95)α1- COO^−^/AsnH33 ND (3.08)α1- COO^−/^HisH35 NE (2.68)α1- COO^−/^GlyH96 NH (3.05)α1- COO^−/^GlyH100a NH (2.82)	276.65	TyrL32, AsnL34, TrpL91,AlaL93, AsnL94, LeuL96,AsnH33, HisH35, ValH50IleH51, TyrH52, AspH56,TyrH57, SerH58, GlyH95,GlyH96, AlaH97, GlyH100a,IleH100b, MetH100c	3.02	C38– SerH58HB (1.89)C64– TrpL91H (2.3)C64– TrpL91C (3.1)	4.62	1.62
	αα–Fe(DoCPP)	133.4	42.01	−45.26	3.19	α1- COO^−/^AsnH33 ND (2.97)α1- COO^−/^HisH35 NE (2.88)α1- COO^−/^GlyH96 N (2.87)α1- COO^−/^GlyH100a N (2.9)α2- COO^−/^AlaH97 N (3.7)	248.9	TyrL32, TrpL91, TyrH52,AsnH33, HisH35, ValH50,IleH51, AspH56, TyrH57,SerH58, GlyH95, GlyH96,AlaH97, GlyH100a, IleH100b	0.87	C70– GlyH100aHA(1.97)	2.38	0.83
	αβ–Fe(DoCPP)	125.87	40.51	−43.79	2.67	α- COO^−/^AsnH33 ND (3.06)α- COO^−/^HisH35 NE (2.85)α- COO^−/^GlyH96 N (2.85)α -COO^−/^GlyH100a N (2.6)	251.09	TyrL32, AsnL34, TrpL91,TyrH52, AsnH33, HisH35,ValH50, IleH51, AspH56,TyrH57, SerH58, GlyH95,GlyH96, AlaH97, GlyH100a,IleH100b	0.92	C58(H) – SerH58HB2 (2.15)	2.36	0.74
	Fe(MoCPP)	126.91	42.1	−43.84	2.64	COO^−/^AsnH33 ND (3.04)COO^−/^HisH35 NE (2.67)COO^−/^GlyH100a N (2.73)	252.46	TyrL32, AsnL34, TrpL91,TyrH52, AsnH33, HisH35,ValH50, AspH56, TyrH57,SerH58, GlyH95, GlyH96,AlaH97, GlyH100a, IleH100b	0.29	–	1.46	1.75
14H7	Fe(ToCPP)	101.53	31.54	−35.62	3.26	α1-COO^−/^TYRH52 OH (2.63)α2-COO^−/^TYRH32 OH (2.96)α2-COO^−/^SERH97 OH (2.96)β- COO^−/^ASNL94 ND (3.05)	164.57	TyrL32, TrpL91, AsnL94,TyrH32, AsnH33, HisH35,TyrH52, AsnH54, AspH56,ThrH57, SerH58, GlyH100a,LeuH100b	0.22	–	3.87	TyrH32 OH (4.5)
	αα–Fe(DoCPP)	116.34	29.84	−32.08	1.98	α1-COO^−/^SERH97 OG (2.58)α2-COO^−/^TYRH32 OH (2.76)	170.96	TyrL32, TrpL91, TyrH32,TyrH52, AspH56, GlyH100a,LeuH100b	0.12	–	2.12	TyrH32 (4.01)
	αβ–Fe(DoCPP)	102.66	29.43	−31.64	1.99	α–COO^−/^TYRH32 OH (2.73)α–COO^−/^SERH97 OG (2.80)	166.85	TyrL32, TrpL91, AsnL94,TyrH32, TyrH52, AsnH54,AspH56, ThrH57, SerH58,GlyH100a, LeuH100b	0.19	C70– SERH58 OH (2.1)	2.03	TyrH32 (4.13)
	Fe(MoCPP)	109.55	30.63	−32.17	1.88	COO^−/^TYRH52 OH (2.6)	174.54	SerL30, TyrL32, TrpL91,TrpL93, TyrH52, SerH97,GlyH100a.	0.15	–	1.4	TyrL32 OH (2.30)

1 The buried surface area was obtained by subtracting the molecular surfaces (calculated using the UCSF Chimera environment) of the nonbonded cofactor and of the antibody alone, from that of the complex and by dividing the result by 2.

2 The ChemScore scoring is defined as: Score = −(ΔG+S_clash_+S_internal_) where the total free energy change that occurs upon ligand binding ΔG = −5.4800 −3.3400*S_hbond_ −6.0300*S_metal_ −0.1170*S_lipo_ +2.5600*H(rot). S_internal_ is the energy term for the internal rotations of the cofactor, S_metal_ that for the metal interactions and H_rot_ that for the frozen rotable bonds.

3 For 13G10, distance between the iron atom in the modeled complex and Mg^2+^ in the crystal structure. For 14H7, distance to iron.

In the most stable 13G10/Fe(ToCPP) predicted complex, the hydrophobic CDRH3 loop stacks against the most buried carboxyphenyl group of the porphyrin hapten (Figure 5A) and must therefore have been specifically selected by the immune system. Ligand recognition is achieved through H-bonds (Figure 5A), van der Waals contacts and stacking interaction (Table 3 and Figure 6A). In the calculated complex, the metal of the porphyrin is located close to the magnesium atom that has been characterized in the X-ray structure (Figure 3B and 4A, Table 3). In the 20 first solutions with low energy for the 14H7/Fe(ToCPP) complex, the porphyrin is always located in the solvent-exposed region of the binding site (Figure 5E and 6E). This leads to very variable orientations of the cofactor with weaker complementarity to the antibody than in 13G10. The best solution obtained for 14H7 is 10 kJ/mol less stable than that for 13G10 because of more extensive hydrophobic interactions in the case of 13G10 (Table 3). Indeed, while the hydrogen bonding energy is the same for the two complexes, the lipophilic energy is about 100 kJ/mol lower in 13G10. Moreover, less clashes can be observed in 14H7, compared with 13G10. These observations are consistent with a lesser degree of complementarity between the cofactor and the antibody in 14H7 than in 13G10. In the former, the cofactor remains solvent exposed, with the most important interactions taking place with TyrH32, TyrH52 and SerH97, while in the latter, the cofactor binds more deeply inside the cavity with the H-bonding interactions taking place with more buried residues: AsnH33, HisH35 and the NH groups of GlyH96 and GlyH100a.

Our models of the complexes of 13G10 and 14H7 with Fe(ToCPP) indicate that no residue in the vicinity of the cofactor is able to chelate the metal in either antibody. The nearest residue (AsnH33 in 13G10 and TyrH32 in 14H7) has its Nδ or OH atom 4 Å and 4.5 Å away, respectively, from the metal. Apparently, the steric hindrance caused by the carboxyphenyl groups prevents the iron from approaching any residue of the protein.

### Rationalization of the Binding of Alternative Cofactors

To obtain better mimics of peroxidases, capable of binding one imidazole ligand, complexes of 13G10 and 14H7 with less hindered iron(III)-tetraarylporphyrins, bearing only one or two carboxyphenyl substituents (monosubstituted Fe(MoCPP) and α,β or α,α, di-substituted Fe(DoCPP)), were designed (Figure 1) [16]. Although all 13G10/porphyrin complexes were shown to bind only one imidazole ligand, the affinity for imidazole of the α,α and α,β-Fe(DoCPP) complexes was 2–3 fold lower than that of 13G10/Fe(ToCPP). Interestingly, the 13G10/dicarboxyphenyl porphyrin complexes presented higher activity for ABTS oxidation than the original Fe(ToCPP)/13G10 complex in the presence of 50 mM imidazole. The crystal structures provided in this work combined with molecular modeling offer the opportunity to rationalize such findings.

**Figure 7:**
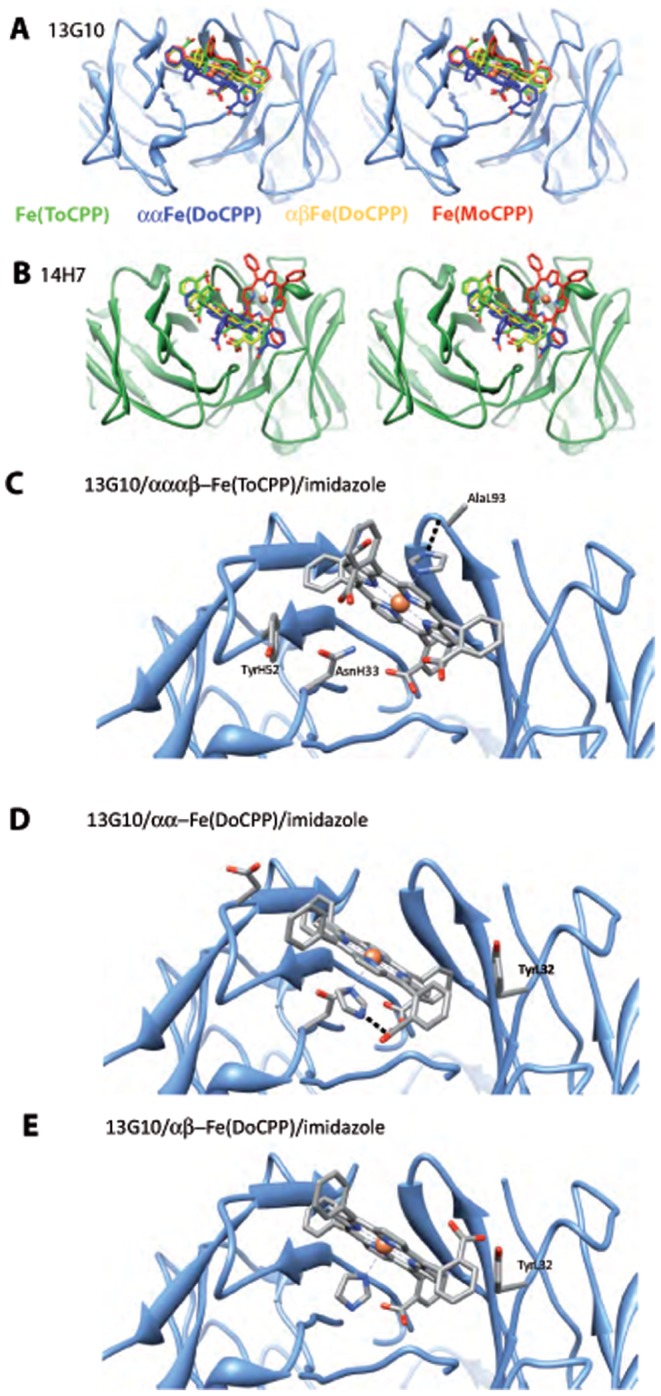
Comparison of the binding of the different porphyrins to 13G10 and 14H7 and models of imidazole binding to the 13G10/porphyrin complexes. Stereoviews of the superposition of the different porphyrins in the combining sites of 13G10 (**A**) and 14H7 (**B**). Fe(ToCPP), αα-Fe(DoCPP), αβ-Fe(DoCPP) and Fe(MoCPP) are colored green, blue, yellow and red, respectively. For 13G10, the porphyrins of Fe(MoCPP), αα-Fe(DoCPP) and αβ-Fe(DoCPP) superimpose onto that of Fe(ToCPP)(without taking into account the carboxylate substituents) with rmsds of 0.5, 3.0 and 1.7 Å^2^, respectively. For 14H7, the porphyrins of Fe(MoCPP), αα-Fe(DoCPP) and αβ-Fe(DoCPP) superimpose onto that of Fe(ToCPP) with rmsds of 9.1, 3.1 and 0.9 Å^2^, respectively. **C** Model of imidazole bound to 13G10/Fe(ToCPP). The binding of the imidazole molecule is favored by an H-bonding interaction with the main chain of AlaL93. **D** Model of imidazole bound to 13G10/αα-Fe(DoCPP). Despite some clashes, imidazole binding is stabilized by H-bonds with AsnH33 and one of the buried carboxylates. Stabilization is also achieved by hydrophobic contacts as imidazole binds in the cavity left between the cofactor and the protein. **E** Model of imidazole bound to 13G10/αβ-Fe(DoCPP). Imidazole is located on the buried side of the porphyrin but does not make any interaction with the protein, the β-carboxylate being oriented toward the solvent.

First, the mono and disubstituted porphyrins were docked into the combining sites of the two antibodies (Figure 5B–D, 6B–D, 7A and B, Table 3). Overall, the four porphyrins have relatively similar binding modes to 13G10, with a good complementarity of a major part of the macrocycle with the binding site (Figure 6A–D and 7A). A strong interaction between one of the porphyrin carboxylates and both AsnH33 and HisH35 of CDRH1 is always present and appears as the most important feature in the recognition of the cofactors by 13G10. This is sustained by the fact that the complexes with Fe(ToCPP) and Fe(MoCPP), despite differing by three carboxylates and making five and three H-bonds with 13G10, respectively, have similar binding energies (Table 3). Thus, increasing the number of carboxylates in the cofactor does not mean an overall better binding because it also increases steric hindrance and results in more important bad contacts (Table 3). Slight differences are still observed in the way the ligands penetrate into the cavity, with αα-Fe(DoCPP) displaying the highest buried surface, the lowest one being obtained for Fe(ToCPP).

Fe(MoCPP) binds to 14H7 in a very different way compared with Fe(ToCPP) (Figure 7B). For αβ-Fe(DoCPP), the binding mode to 14H7 is very similar to that of Fe(ToCPP), while for αα-Fe(DoCPP), the porphyrin is noticeably displaced. However, overall, the binding modes of the three alternative cofactors are similar in energy, including for its individual terms (Table 3).

Docking imidazole in the various porphyrin/13G10 complexes indicates that two molecules of imidazole can bind the iron, one on each side of the cofactor. However, imidazole binds preferentially on opposite faces of the porphyrin, depending on the cofactor (Figure 7C, D and E). For Fe(ToCPP), imidazole binds to the solvent-exposed face, whereas in the case of the dicarboxylates-containing porphyrins, it binds in the cavity formed between the cofactor and the protein. The overall binding energies are very similar and the main differences appear in the hydrogen bond and lipophilic terms (Table 4).

**Table 4:** Statistical analysis of the lowest energy structures for the docking of imidazole in the 13G10/Fe(ToCPP) and 13G10/Fe(DoCPP) models.

Antibody	Ligand	Score	ΔG_binding_ (kJ/mol)	S_hbond_	Hydrogen bondingInteractions(distances in Å)	S_metal_	S_lipo_	S_clash_	Clashes (distancesin Å)
13G10	Fe(ToCPP)	22.61	−23.08	0.98	AlaL93-O (2.64)	0.9	76.42	0.47	–
	αα–Fe(DoCPP)	21.54	−22.87	0.63	COO^−^ (2.65)	0.81	88.96	1.33	AsnH33 (2.45)
					AsnH33 ND (2.96)				Fe (2.62)
	αβ–Fe(DoCPP)	19.88	−19.93	0.0	–	0.9	77.21	0.05	–

### Structural Basis of Catalysis: Comparison to the Ferrochelatase and Porphyrin-dependent Peroxidase Antibody 7G12

**Figure 8:**
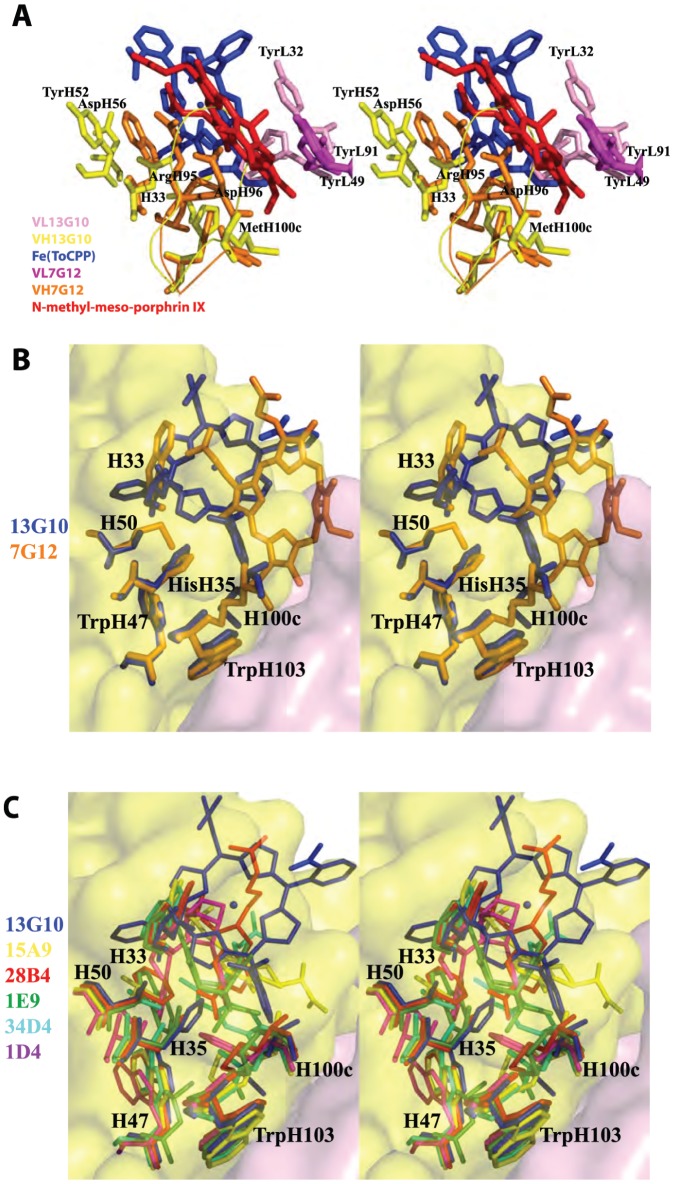
Comparison of Fab 13G10 with other catalytic antibodies. **A** Comparison of the combining sites of the two porphyrin-dependent peroxidase antibodies 13G10 and 7G12. The α-carbons of the framework of the heavy chain variable domain of 7G12 (in orange) have been superimposed on those of 13G10 (in yellow), which results in a large variation in the positions of the light chain variable domains (VL 7G12 in magenta and VL 13G10 in pink). The rms deviation of 0.964 Å reflects the different packing of the VH and VL domains in λ-light chain antibodies (13G10) and κ-light chain antibodies (7G12). The hapten of 13G10 is represented in blue and that of 7G12 in red. **B** Comparison of the cavity deepness of antibodies 13G10 (in blue) and 7G12 (in orange). A section of the molecular surface of the combining site of antibody 13G10 is colored in yellow for the heavy chain and pink for the light chain. **C** Comparison of the cavity shapes of the nonhydrolytic catalytic antibodies with a bulky residue at position preceding H101. 15A9 (yellow), PDB code 2BMK; 28B4 (red), PDB code 1KEL; 1E9 (green), PDB code 1C1E; 34D4 (cyan), PDB code 1Y18; 1D4 (magenta), PDB code 1JGU.

The structure of 13G10 can be compared with that of the κ-light chain metallochelatase and porphyrin-dependent peroxidase antibody 7G12 (Fig. 8A and B). Antibody 7G12, induced against a distorted *N*-methylmesoporphyrin IX, a transition state analogue for porphyrin metalation [48], catalyzes the chelation of various metals by mesoporphyrin IX. In addition, the complex of 7G12 with Fe(III)-mesoporphyrin IX was shown to catalyze the oxidation of several chromogenic peroxidase substrates by hydrogen peroxide with more than 200 turnovers with *k*_cat_ of 6 s^−1^ and *k_cat_/K_m_* of 300 M^−1^ s^−1^ [49]. Thus, the peroxidase activity of 7G12 is similar to that of 13G10. The crystal structure of antibody 7G12 complexed with *N*-methylmesoporphyrin IX has shown that the antibody induces geometric strain in the porphyrin substrate to catalyze porphyrin metalation [50], [51], [52]. The carboxylate side-chain of AspH96 of 7G12, which is positioned 1.9 Å from the center of the porphyrin ring (Fig. 8A), is thought to act as a catalytic residue in the metal chelatase reaction by deprotonating the substrate and chelating the metal [50]. For the peroxidase reaction, AspH96 is too near to the center of the porphyrin ring to act as a distal ligand but it is in an appropriate position to act as a proximal ligand for the iron atom of Fe(III)-mesoporphyrin IX. Hydrogen peroxide is expected to approach the non-obstructed side of the porphyrin ring, opposite to AspH96, which is surrounded only by hydrophobic residues such as TyrL49 and TyrL91. The active site of 7G12 does not reveal the presence of distal ligands susceptible to enhance the peroxidase activity of Fe(III)mesoporphyrin IX.

Directed and random mutagenesis were used to generate libraries of 7G12, and antibodies with increased peroxidase activity were selected by phage display using an activity-based strategy [53]. A mutant, in which TyrL49 was replaced by Trp, had a 10-fold increase in *k*_cat_/*K*_m_. In addition, two mutants bore mutations thought to affect the packing between the porphyrin ring and TyrL49 or TyrL91. Because TyrL49 and TrpL91 are involved in π-stacking interactions with the porphyrin ring in the 7G12/*N*-methylmesoporphyrin IX complex (Fig. 8A), it was proposed that the mutations could help to stabilize the radical cation on the porphyrin ring and yield to higher peroxidase activity [53].

In 13G10 and 7G12, a bulky residue preceding residue H101 (Met) is positioned at the bottom of the active site cavity, which leads to shallow binding sites (Fig. 8). This positions the porphyrin haptens at the surface of the combining site, contacting almost exclusively residues of the CDRs. The larger elbow angle in λ-light chain antibody 13G10 coupled with a longer CDRH3 (H95–H101) results in a shallower binding site in 13G10 compared with 7G12 (Fig. 8A and B). In contrast to 7G12, no residue is correctly positioned to bind the iron in the 13G10-Fe(ToCPP) complex.

## Discussion

Antibodies elicited against carefully designed transition state analogues have been reported to catalyze a wide range of chemical reactions [54], [55], [56], [57], [58], [59]. However, the crystal structures of these antibody catalysts have revealed that most antibodies generally act by simple transition state stabilization [58], [60], [61] and only a few utilize covalent chemistry [62], [63], [64], [65]. The incorporation of cofactors has been proposed as a possible strategy to expand the catalytic scope of antibodies, in particular to lead to redox catalysis [56]. Actually, the combination of the intrinsic reactivity of a cofactor with the tailored binding specificity of an antibody has been underutilized. However, cofactor and antibody effectively complement each other: like in enzymatic catalysis, the antibody enhances the catalytic efficiency of the cofactor and ensures reaction specificity, stereospecificity and substrate specificity. Several antibodies raised against a porphyrin hapten and possessing catalytic activity have been reported [12], [48], [49], [66], [67], [68], [69], [70], [71], [72]. Moreover, catalytic antibody-cofactor complexes have been structurally characterized for a periodate-dependent oxygenation catalyst [73], [74], and a pyridoxal-5′-phosphate-dependent antibody that catalyzes the transamination of D-amino acids [75].

Antibodies 13G10 and 14H7, induced against Fe(ToCPP), were shown to display peroxidase activity in the presence of iron-porphyrin cofactors. Biochemical studies had already given insights into the interactions between the antibodies and the cofactor. First, UV-visible studies have shown that the binding of the porphyrin into the antibodies is not accompanied by a change of the high spin state of the iron(III) and that the porphyrin binds in a hydrophobic pocket [13]. Moreover, the antibodies possess similar affinities for the metallated or non-metallated cofactor (*K_d_* = 3–5 nM) [14]. Altogether, these results indicated that no amino acid binding the iron atom had been induced in the antibody combining sites by the Fe(ToCPP) hapten. The determination of the apparent dissociation constants for the variously substituted porphyrins by competitive ELISA indicated that the antibodies do not bind tetraphenylporphyrin and allowed a model to be proposed, where two thirds of the porphyrin macrocycle could be inserted in the binding pocket, with two carboxylates in α,β positions being more specifically bound to the protein [14]. Tetraaryl porphyrins bearing only one *meso*-ortho-carboxyphenyl substituent could still bind 13G10 and 14H7, although with a 50-fold reduction in affinity [14]. Finally, absorption spectroscopy studies have shown that, whereas the iron(III) of Fe(ToCPP) is able to bind two imidazole ligands, the Fe(ToCPP)-13G10 complex can fix only one, which inhibits its peroxidase activity [16].

All catalytic antibodies, whose crystal structure had been solved until now, belonged to the 95% mouse antibodies that possess a κ-light chain [76], except one [77]. Many of these catalytic antibodies share a deep combining site formed not only by residues of the CDRs but also by residues of the framework (Figure 8C) [58], [61]. This observed structural convergence was due in part to the use of similar hydrophobic haptens to induce the antibodies. 13G10 and 14H7, elicited against a very different iron-porphyrin hapten, belong to the IgG1, λ class. As observed for other catalytic antibodies [78], the mature genes of 13G10 and 14H7 display a high conservation degree with their germline counterparts.

Among the few examples of crystal structures of murine Fabs with λ-type light-chain that have been solved, ten of them are those of Fabs complexed with small haptens [22], [79], [80], [81], [82], [83], [84], [85] (Table 2) and several of them with a peptide or protein antigen [86], [87], [88], [89], [90], [91], [92], [93]. These crystal structures have shown that the combining site of λ-light chain antibodies is formed by different amino acid residues than that of κ-light chain antibodies (Compare Table 2 with Table 2 of reference [61]). In particular, the X-ray structures of antibodies of different isotypes (λ– and κ– type) that bind to the same hapten molecule have revealed drastically different binding modes of the ligand [83], [84], [94]. It was therefore anticipated that catalytic antibodies 13G10 and 14H7 will possess a combining site different from that of previously crystallographically characterized κ–light chain catalytic antibodies and it was interesting to understand how different they were.

The comparison of the cavity shapes of non hydrolytic catalytic antibodies led to separate them into two categories, depending on the nature of the residue preceding H101 [61]. Antibodies possessing a small residue (ie. Gly, Ser) had a common deep combining site, whereas those with a bulky residue (Phe, Met) displayed shallow cavities with very different shapes. Antibodies 13G10 and 14H7 differ at this position, possessing Met and Leu, respectively, and their structures reveal that their combining site cavities is not very deep. In addition, their combining sites lie more on the protein surface, compared with the other catalytic antibodies possessing a bulky residue preceding H101 (Figure 8C). Because CDRH3 that is composed of several glycines and residues with small side chains is predicted to be flexible, the combining sites of the antibodies 13G10 and 14H7 do not share a high similarity in shape although the two antibodies share a high sequence similarity. Indeed, the deeper cavity observed in the structure of 13G10 compared with 14H7 comes mainly from the different conformations of CDRH3.

The structures of 13G10 and 14H7 indicate that the amino acids prone to bind the ligand are the same as in the other λ-light chain antibodies (Table 2), with only a few residues that belong to the L chain taking part in the combining site, and CDRH2 making an important contribution. About half of the hapten appears to be buried in the combining sites of 13G10 and 14H7, with CDRH3 and residues TyrL32, TyrH52 and TrpL91 stacking against the porphyrin ring (Figure 6). Although this accounts for the nM affinity of the hapten for the antibodies, this is lower than the two thirds predicted from the biochemical data [14], presumably because induced fit is expected to occur in the complexes, which is not taken into account in the calculated interactions. Molecular docking predicts important differences for the recognition of Fe(ToCPP) by the two antibodies. The shallower and wider binding site of 14H7, as observed in the crystal structure, does not allow the hapten to bind as deeply as it does in 13G10. Similar results were obtained for the docking of the other cofactors (Figure 7A and 7B).

Like in peroxidases, the heme environment of 13G10 and 14H7 consists of numerous hydrophobic residues. In heme peroxidases, the iron atom is bound to the apoprotein by the proximal histidine. This axial ligand was shown to be important for modulating the redox potential of the iron and thus, the catalytic activity of the enzyme [95]. However, our docking models indicate, in agreement with the biochemical data, that no amino acid residue that binds the iron has been induced in the combining sites of 13G10 and 14H7. The key step of the mechanism of peroxidases is usually the cleavage of the O–O bond of H_2_O_2_ or ROOH, with the release of a water molecule and formation of the highly reactive iron (V)-oxo intermediate, assisted by the distal histidine and an arginine. However, this function is fulfilled by a catalytic glutamate base positioned 4.9 Å apart from the iron for a direct attack in *Caldariomyces fumago* chloroperoxidase [96]. Neither induction by the Fe(ToCPP) hapten itself, nor the use of differently substituted porphyrin cofactors, led to the positioning of any histidine or arginine residue in 13G10 or 14H7 that could participate in the heterolytic cleavage of the O-O bond of peroxide, like in peroxidases. However, previous biochemical results suggested that a carboxylic acid side chain of antibody 13G10 could participate in catalysis by protonating one of the oxygen atom of H_2_O because it was shown that k_cat_ increases sharply below pH 5 for the 13G10-Fe(ToCCP) complex [15]. However, no COOH side-chain of the 13G10 antibody is correctly localized to act as a general acid-base catalyst. Indeed, the only carboxylic acid side-chain in the combining site of 13G10 that could play a role in catalysis belongs to AspH56 (Table 3, Fig. 3C, 4C, 5, 6 and 8A). However, its nearest oxygen atom is positioned 10.6 Å away from the iron, a distance that necessitates a mediating water molecule for AspH56 to act as a general acid catalyst in the peroxidase reaction. Acid catalysis by the ortho carboxylate group of iron (II) [*meso*-(ortho-carboxyphenyltriphenyl-porphyrin has previously been proposed in aldoxime dehydration *via* the protonation of the substrate hydroxy group [97]. Thus, one of the two buried carboxylate groups of the hapten could fulfill the same function in the 13G10-Fe(ToCCP) complex. Our models of the porphyrin-13G10 complexes reveal that, in addition to them, the polar residues best positioned to play a role in catalysis are AsnH33 and TyrH52, also located on the buried side of the porphyrin (Figure 5 A–D). H_2_O_2_ could bind on the same sheltered face of the porphyrin ring, in a hydrophobic pocket similar to that present in horseradish peroxidase [98]. This location of the binding site of hydroperoxide would explain the remarkable thermostability of the cofactor-antibody complex and the multiple turnovers of the reaction. In cytochrome *c* oxidase, the radical cation in the iron(IV)oxoporphyrin intermediate was delocalized onto the indole ring of TrpL91 [8]. Similarly, it was proposed that TyrL49 and TyrL91 that stack against the porphyrin ring of the hapten could fulfill a similar function in catalytic antibody 7G12 and enhance the peroxidase activity of the 7G12/Fe(III)mesoporphyrin complex [53]. In the models of 13G10 and 14H7 with the different porphyrins, TrpL91, TyrL32 and TyrH52, which are predicted to stack against the porphyrin ring (Fig. 6), could act in the same way.

Imidazole was used as an iron ligand that might mimic the proximal histidine in the 13G10/porphyrin complexes. Molecular docking indicates that imidazole preferentially binds on opposite faces of the porphyrin ring in the 13G10/Fe(ToCPP) and 13G10/Fe(DoCPP) complexes (Figure 7C to E). Imidazole is predicted to bind to the solvent-exposed face of Fe(ToCPP), which might hinder binding of the hydroperoxide substrate to the sheltered face of the cofactor and lead to the inhibition of the peroxidase activity. In contrast, imidazole is predicted to bind in the cavity formed between 13G10 and αα- or αβ-Fe(DoCPP) (Figure 7D and E), which likely represents a higher affinity site and would explain that the affinity for imidazole of the α,α- and α,β-1,2-Fe(DoCPP) complexes was 2–3 fold lower than that of 13G10/Fe(ToCPP). In this manner, imidazole would be located appropriately to act as the proximal histidine. In this case, hydroperoxide would bind on the solvent-exposed face of the porphyrin and TyrL32 could assist in the cleavage of the O-O bond of hydroperoxide by functioning as an acid catalyst. In the case of αβ-Fe(DoCPP), the β carboxylate of could also play this role. This would account for the 8–9–fold enhancement of the catalytic efficiency of the peroxidase reaction in the presence of 50 mM imidazole, when 13G10 is complexed with the di-substituted cofactors compared with Fe(ToCPP).

In the future, better catalysts could be obtained by directed mutagenesis of 13G10 and 14H7. Replacing AsnH33 by a histidine in 13G10 could lead to the binding of its imidazole group to the iron atom of Fe(ToCPP), which could enhance the peroxidase activity of the 13G10/porphyrin complex. Indeed, antibodies induced against microperoxidase 8 that were shown to possess an axial histidine coordinating the iron atom had a better peroxidase activity than 13G10 and 14H7 [99], [100]. Mutating TyrL32 to histidine or arginine to generate a better acid catalyst or an amino acid that enhances the polarization of the O-O bond could also increase the catalytic activity of the 13G10/Fe(DoCPP)/imidazole complexes. Alternatively, antibodies with enhanced peroxidase activity could be obtained by phage display using an activity-based strategy for selecting oxidative catalysts, as described previously [53].

### Conclusion

Understanding the structure-function relationship of catalytic antibodies with a λ-light chain is important to shed light on the diversity of catalytic antibodies, which may help to broaden the scope of these catalysts. The anti-porphyrin antibodies 13G10 and 14H7, with a λ-light chain, were shown to possess shallow hapten binding pockets compared with the other structurally characterized catalytic antibodies. The structural complementarity of the Fe(ToCPP) cofactor to the hydrophobic binding pocket of antibodies 13G10 and 14H7 leads to a remarkable thermostability of the cofactor-antibody complexes and allows multiple turnovers of the peroxidase reaction. Molecular modeling indicates that the recognition of various porphyrins with different carboxyphenyl substituents is achieved mainly by stacking interactions but also by crucial hydrogen bonds with two or three carboxylate groups. Our models explain why one carboxyphenyl substituent is sufficient for a good affinity of the porphyrin cofactor for 13G10 and 14H7. CDRH1 and CDRH3 appear to play key roles for binding Fe(ToCPP) and no proximal ligand of the iron was induced in 13G10 and 14H7. The increase of the peroxidase activity of the cofactor, when bound to the antibodies, could be explained by a loss of entropy due to accessibility of H_2_O_2_ to only one of the two faces of the porphyrin ring, and possibly by the stacking of several aromatic groups onto the porphyrin ring that would stabilize the radical cation in the iron(IV)oxoporphyrin intermediate in the peroxidase reaction. Moreover, one of the buried carboxylic group of the porphyrin substituents may also participate as a general acid catalyst, with the implication that H_2_O_2_ would bind on the sheltered face of the porphyrin ring. The carboxylic group would facilitate the heterolytic cleavage of the O-O bond, with the release of a water molecule and formation of the highly reactive iron (V)-oxo intermediate. Interestingly, an imidazole ligand that mimics the proximal histidine in peroxidases can be modeled in the complexes of 13G10 with αα-Fe(DoCPP) and αβ-Fe(DoCPP) on the sheltered face of the porphyrin ring, with TyrL32 correctly positioned to act as an acid catalyst, in agreement with these complexes showing higher catalytic efficiency compared with Fe(ToCPP), in the presence of imidazole. The similar binding energies of 13G10 and 14H7 for the alternative cofactors and the original hapten open novel possibilities for developing other porphyrinic-based cofactors for these antibodies. In future work, in order to obtain catalytic antibodies that mimic cytochromes P450, one should design a substituted porphyrin hapten that generates binding sites for the substrate in addition to that of the metalloporphyrin in the antibodies.

#### Data deposition

The atomic coordinates and structure factors of Fab13G10 and Fab14H7 have been deposited at the Protein Data Bank (PDB codes 4amk and r4amksf, and 4at6 and r4at6sf, respectively).

## Supporting Information

Figure S1 **Detection of twinning and determination of the twin fraction in the 14H7 crystals. A** Estimation of the twin fraction α by Britton plot analysis. The percentage of negative intensities after detwinning is plotted as a function of the assumed value of α. The estimated value of α is extrapolated from the linear fit (green line). **B** Estimation of the twin fraction α using the H*-*plot. The cumulative fractional intensity difference of acentric twin-related intensities H {H = |*I*(*h* 1) − *I*(*h* 2)|/[*I*(*h* 1) + *I*(*h* 2)]} is plotted against H. The initial slope (green line) of the distribution is a measure of α.
(DOCX)
