# Supplementary figures and images for: Crystal Structure of Two Anti-Porphyrin Antibodies with Peroxidase Activity
Source: PLoS One. 2012 Dec 11;7(12):e51128. doi: 10.1371/journal.pone.0051128 (PMC3519839; doi:10.1371/journal.pone.0051128)

**Supplementary Figure 1**

**A**


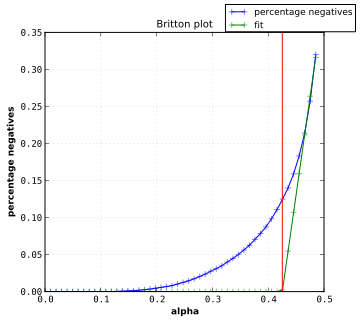


**B**


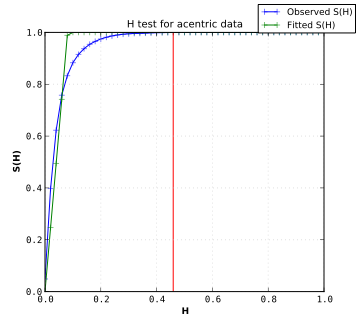

Supplement: Figure S1 — Detection of twinning and determination of the twin fraction in the 14H7 crystals. A Estimation of the twin fraction α by Britton plot analysis. The percentage of negative intensities after detwinning is plotted as a function of the assumed value of α. The estimated value of α is extrapolated from the linear fit (green line). B Estimation of the twin fraction α using the H-plot. The cumulative fractional intensity difference of acentric twin-related intensities H {H = |I(h 1) − I(h 2)|/[I(h 1) + I(h 2)]} is plotted against H. The initial slope (green line) of the distribution is a measure of α. (DOCX) [file pone.0051128.s001.docx]
